# Properties for Sourcing Nigerian Larvicidal Plants

**DOI:** 10.3390/molecules19068363

**Published:** 2014-06-19

**Authors:** Adeleke Clement Adebajo, Funmilayo Gladys Famuyiwa, Fatima Abosede Aliyu

**Affiliations:** Department of Pharmacognosy, Faculty of Pharmacy, Obafemi Awolowo University, Ile-Ife 220282, Osun State, Nigeria; E-Mails: caadebajo@yahoo.com (A.C.A.); fatmababe@yahoo.com (F.A.A.)

**Keywords:** methanolic extracts, nigerian medicinal plants, *Aedes aegypti*, plant larvicides

## Abstract

*Aedes aegypti* is the primary vector of chikungunya, yellow and dengue fevers. Dengue fever is the major cause of child morbidity and hospitalisation in some Asian and African countries, while yellow fever is prevalent in Nigeria. The development of resistance to the available insecticides has necessitated the continued search for safer ones from plants. Eighteen plant extracts with ethnomedical claims of or demonstrated febrifuge, antimalarial, insecticidal and insect repellent biological activities were tested for activity against the fourth instar larvae of *Aedes aegypti.* About 61% of the eighteen extracts demonstrated high to moderate larvicidal activity. Extracts of *Piper nigrum* and *Abrus precatorius* seeds were the most active and the larvicidal constituent(s) of the latter should be determined.

## 1. Introduction

*Aedes aegypti* Linn. (Culicidae) is the primary vector of chikungunya, yellow and dengue fevers [1]. Dengue fever is the major cause of child morbidity and hospitalisation in some Asian and African countries, while yellow fever is prevalent in Nigeria as well as other tropical countries. The absence of established diagnostic facilities has hindered the detection of dengue virus in Nigeria, as its symptoms have been often mistaken with malaria, thyphoid, *etc.* [2]. The conventional insecticides used in mosquito control are limited, with reported adverse effects on the ecosystem. Also, these vectors have developed resistance to the available insecticides [3]. These problems have therefore necessitated the continued search for safer insecticides in eradication or reduction of the vectors’ populations. Since they are highly biodegradable, well tolerated by the ecosystem and have low mammalian toxicity, medicinal plant extracts have been reported as safer alternatives [4]. More successes have been reported with plants tested for activities suggested by their ethnomedicinal/folkloric uses [5,6,7], a statement agreed to by the WHO [8]. Also, the larvicidal, pupicidal and adulticidal activities of many plant constituents were found comparable to those of the standard drugs [4,9,10,11,12,13] Since larvicidal activity is not traditionally known, it was suggested that ethnomedical claims or febrifuge, antimalarial, insecticidal and insect repellent biological activities could be considered as part of the factors for sourcing plant larvicides [14]. Hence this study is a further attempt in testing this hypothesis by assessing the larvicidal activity of an additional fourteen plants, listed in Table 1, against fourth instar larvae of *A.* *aegypti.*

## 2. Results and Discussion

Dead larvae were those that could not be induced to move when probed with a needle in the siphon or the cervical region while moribund larvae were those incapable of rising to the surface or not showing the characteristic diving reaction when the water was disturbed [15]. According to WHO [15], the percentage mortality should be calculated by adding the moribund larvae to those that are dead. However, similar to an earlier work [14], the larvae that were moribund after 48 h were added to the living, hence a more stringent assay of larvicidal activity with higher LC_50_ and LC_90_ values for the extracts. The LC_50_ values of extracts of the leaves of *L. owariensis*, *C. indica*, *C. patens*, *H. opposita*, *M. indica* and *A. boonei*, woods of *A. altilis* and *E. heterophylla*, *E. chlorantha* stem bark, and leaf and stem of *C. afer* at 24 h were significantly higher than those at 48 h, similar to their LC_90_ values (Table 2). This showed that longer exposure to these extracts benefitted their larvicidal activities, probably indicating cumulative effects in their activities. On the other hand, the LC_50_ values of extracts of the seeds of *P. nigrum* and *A. precatorious*, *C. longa* rhizome, *A. altilis* stem bark, whole plant of *S. biafrae*, leaves of *D. cumminsii* and *M. koenigii* as well as that of Endosulphan at 24 h were comparable with those at 48 h, similar to their LC_90_ values at 24 and 48 h (Table 2). This may indicate short larvicidal activities of these extracts [14,16]. Extracts with LC_50_ < 2 mg/mL were regarded as very active [14]. Therefore, LC_50_ of 0.01 and 0.85 mg/mL at both 24 and 48 h for *P. nigrum* and *A. precatorius* seed extracts, respectively qualified them as the most active of the eighteen extracts tested. Endosulphan, a commercial insecticide, with LC_50_ of 0.93 and 0.90 mg/mL at 24 and 48 h, respectively had larvicidal activity that is comparable (*p* > 0.05) to those of *P. nigrum* and *A. precatorius* (Table 2). Insecticidal activity has been reported for these two plants [17,18], which are also either used ethnomedicinally to treat malaria or fever (Table 1), making them possible good plant larvicides and sources of larvicidal compounds. This result therefore lends credence to the hypothesis proposed for sourcing plants larvicides [14].

**Table 1:** List of plants used.

Name of Plant	Family	Part	Relevant Use/Activity	Collection Place	Ref.
*Abrus precatorius* L.	Fabaceae	Seed	Antimalarial	Uyo	[14]
*Alstonia boonei* De Wild	Apocynaceae	Leaf	Antimalarial	Osun	[19]
*Artocarpus altilis* Forst	Moraceae	Stem bark	Antimalarial	Osun	[20]
*Artocarpus altilis* Forst	Moraceae	Stem wood		Osun	
*Canna indica* L.	Cannaceae	Leaf	Fever, Malaria	Osun	[21]
*Cleistopholis patens* (Benth.) Engl. & Diels	Annonaceae	Leaf	Fever, Antimalarial	Osun	[22]
*Costus afer* K. Schum	Costaceae	Leaf	Antimalarial	Osun	[23]
*Costus afer* K. Schum	Costaceae	Stem		Osun	
*Curcuma longa* L.	Zingiberaceae	Rhizome	Antimalarial	Osun	[21]
*Dioscoreophyllum cummnisii* L.	Menispermaceae	Leaf	None	Osun	*
*Enantia chlorantha* Oliv.	Annonaceae	Stem bark	Fever, Antimalarial	Osun	[24]
*Euphorbia heterophylla* L.	Euphorbiaceae	Wood	Insecticidal	Uyo	[25]
*Hoslundia opposita* Vahl	Lamiaceae	Leaf	Antimalarial	Osun	[26,27]
*Landolphia owariensis* P. Beauv.	Apocynaceae	Leaf	Antimalarial	Osun	[28]
*Mangifera indica* L.	Anacardiaceae	Leaf	Fever	Uyo	[29]
*Murraya koenigii* L.	Rutaceae	Leaf	Fever, Anti-malarial, Insecticidal	Osun	[30]
*Piper nigrum* L	Piperaceae	Seed	Insecticidal, Fever	Uyo	[17]
*Senecio biafrae* L.	Asteraceae	Whole plant	None	Osun	*

*: No reference is given because they do not have any of the prescribed properties.

**Table 2:** Larvicidal activities of the methanolic extracts of some Nigerian medicinal plants against *Aedes aegypti*.

Name of Plant and Part	24 h		48 h	
	LC_50_ *	LC_90_ *	LC_50_ *	LC_90_ *
*Piper nigrum* Seed	0.01 ± 0.12 ^a^	0.02 ± 0.18 ^a^	0.01 ± 0.12 ^a^	0.02 ± 0.18 ^a^
*Abrus precatorius* Seed	0.85 ± 0.00 ^a^	1.37 ± 0.00 ^a^	0.85 ± 0.00 ^a^	1.37 ± 0.00 ^a^
*Curcuma longa* Rhizome	2.62 ± 0.15 ^b^	4.45 ± 0.22 ^b^	2.55 ± 0.15 ^b^	4.41 ± 0.28 ^b^
*Landolphia owariensis* Leaf	3.62 ± 0.15 ^c^	6.11 ± 0.21 ^c^	2.99 ± 0.17 ^c^	5.20 ± 0.20 ^c^
*Canna indica* Leaf	3.84 ± 0.13 ^c^	6.41 ± 0.25 ^c^	2.78 ± 0.11 ^c^	5.09 ± 0.22 ^c^
*Artocarpus altilis* Stem bark	3.90 ± 0.00 ^c^	5.24 ± 0.00 ^c^	3.63 ± 0.68 ^c^	5.10 ± 1.45 ^c^
*Artocarpus altilis* Wood	6.38 ± 0.29 ^e^	10.33 ± 0.22 ^e^	4.41 ± 0.37 ^e^	6.95 ± 0.72 ^d^
*Cleistopholis patens* Leaf	4.41 ± 0.09 ^d^	7.12 ± 0.14 ^d^	3.20 ± 0.24 ^c^	5.30 ± 0.45 ^c^
*Dioscoreophyllum cumminsii* Leaf	4.52 ± 0.03 ^d^	7.48 ± 0.04 ^d^	4.44 ± 0.33 ^e^	6.78 ± 0.66 ^d^
*Enantia chlorantha* Stem bark	4.55 ± 0.69 ^d^	6.48 ± 1.31 ^d^	3.86 ± 0.08 ^d^	5.39 ± 0.09 ^c^
*Hoslundia opposita* Leaf	4.56 ± 0.08 ^d^	7.31 ± 0.19 ^d^	4.05 ± 0.36 ^e^	6.74 ± 0.61 ^d^
*Senecio biafrae* Whole plant	4.73 ± 0.37 ^d^	7.62 ± 1.13 ^d^	4.45 ± 0.21 ^e^	6.85 ± 0.44 ^d^
*Murraya koenigii* Leaf	4.83 ± 0.53 ^d^	6.92 ± 1.05 ^d^	4.38 ± 0.40 ^e^	6.58 ± 0.90 ^d^
*Costus afer* Leaf	8.25 ± 1.15 ^f^	13.54 ± 1.24 ^f^	4.06 ± 0.27 ^e^	6.66 ± 0.51 ^d^
*Costus afer* Stem	9.00 ± 0.29 ^f^	15.00 ± 0.29 ^f^	3.79 ± 0.05 ^d^	5.83 ± 0.19 ^c^
*Mangifera indica* Leaf	8.57 ± 0.59 ^f^	13.77 ± 1.10 ^f^	4.54 ± 0.23 ^e^	7.13 ± 0.42 ^d^
*Euphorbia heterophylla* Wood	IND ^g^	IND ^g^	5.75 ± 0.00 ^f^	8.75 ± 0.00 ^g^
*Alstonia boonei* Leaf	IND ^g^	IND ^g^	IND ^g^	IND ^h^
Endosulphan	0.93 ± 0.06 ^a^	1.61 ± 0.12 ^a^	0.90 ± 0.09 ^a^	1.44 ± 0.11 ^a^

Keys: *: Doses in mg/mL; IND: Indeterminable (no dead larvae at the concentrations tested). Values with different superscripts within columns are significantly different (*p* < 0.05, one-way analysis of variance followed by the Student–Newman–Keuls’ test).

The respective LC_50_ = 0.56 and 0.65 ppm (5.6 and 6.5 × 10^−4^ mg/mL) reported for Aedes albopictus and Culex quinquefasciatus with *P. nigrum* [31] may indicate that these larvae were more susceptible than *A. aegyptii* (Table 2). Furthermore, the LC_50_ of 0.06 and 0.03 mg/mL given for aqueous and ethanolic extracts of its dried ripe fruits against *C. quinquefasciatus*, respectively [32] confirmed that the plant has a high larvicidal activity against many larvae. Piperine has been isolated as the active adulticidal constituent of the plant [33]. After 24 h, the methanolic extracts of *A*. *precatorius* shoot and seeds had LC_50_ of 0.03 and 0.02 mg/mL, respectively against *C. quinquefasciatus* and *Anopheles vagus*, while the ethylacetate extract of its seed had LC_50_ of 0.14 mg/mL against *Culex vishnui* [34,35], indicating that these larvae were more susceptible (Table 2). Additionally, the fruit and seed extracts of *A*. *precatorius* were found toxic to adult mosquitoes [36], suggesting it as a good plant larvicide and adulticide.

The 2.0 < LC_50_ < 4.2 mg/mL given by the extracts of *C. longa* rhizome, *A. altilis* stem bark and leaves of *L. owariensis* and *C. indica* indicated moderate activity while the remaining extracts (LC_50_ > 4.2 mg/mL) were inactive (Table 2). This is the first reported activity of the leaf extracts of *L. owariensis* and *D.* *cumminsii*, leaf and stem of *C. afer*, *A. altilis* stem bark and *S. biafrae* whole plant against any larvae. At 48 h, *C. afer* leaf and stem, *H. opposita* leaf, *C. patens* leaf and *E. chlorantha* stem bark were also moderately active (Table 2), indicating benefit of longer exposure to these extracts and the slow acting nature of their active principles. At 48 h, the larvicidal activities of *M. indica* and *E. heterophylla* were also improved. The *C. longa* rhizome oil (LC_50_ = 0.017 mg/mL) was reported more toxic to *A. gambiae* larvae than the leaf oil (LC_50_ = 0.029 mg/mL) [37]. The lower activity obtained for the extract of this rhizome (Table 2), may indicate that the active constituents were probably more in the volatile oil, although difference in the larvae used may also play a role. Furthermore, its oil hydrolates had LC_50_ of 24.7% and 35.5% v/v against *A. albopictus and C. quinquefasciatus*, respectively [38]. The extract of its rhizome exhibited high but varying activities against the larvae of *Nilaparvata lugens*, *Plutella xylostella*, *Myzus persicae* and *Spodoptera litura*, different species of stored grain pests while the insecticidal principle was identified as *ar*-tumerone [39].

At 24 h, the petroleum ether extract of *C. indica* leaf displayed higher activity (LC_50_ = 0.056, LC_90_ = 0.248 mg/mL) against *C. quinquefasciatus* larvae [40] than the LC_50_ = 3.84, LC_90_ = 6.41 mg/mL obtained for its methanolic extract used in this study (Table 2), indicating differences in the susceptibility of the larvae. The *A. boonei* leaf extract did not kill the larvae after 48 h (Table 2). The aqueous extracts of *A. boonei* stem bark and leaf significantly (*p* < 0.01) reduced the survival and weights of the *Sesamia calamistis* larvae, the pink stalk borer, in a dose dependent manner. Equally high concentrations of the stem bark (2.8% and 2.1%) and leaf (5.6% and 3.5%) have been reported to kill 50% of the larvae at 10 and 20 days after introduction, respectively [41], which would give LC_50_ of 28, 21 and 56, 35 mg/mL for the stem bark and leaf, respectively in 10 and 20 days. The LC_50_ values of 2.70, 11.33 and 12.54 mg/mL given by *A. boonei* leaf extracts, respectively at 24 h against *Anopheles arabiensis* indicated ethanol > aqueous > methanol extracts as the order of larvicidal activity [42]. Similarly, the LC_50_ value of *E. heterophylla* whole plant extract at 24 h could not be determined while its LC_50_ = 5.75 mg/mL at 48 h indicated non-activity (Table 2). Its ethanolic and petroleum ether extracts displayed high activity (LC_50_ = 0.024 and 0.025 mg/mL, respectively) against *Cx. quinquefasciatus* [43], indicating higher susceptibility of this larvae. The inactive methanolic extract of *M. indica* leaf (Table 2) has also been reported to be inactive against *C. quinquefasciatus* [34,44]. The stem bark of *A. altilis* had better activity than its wood while the activity of leaf and stem of *C. afer* was comparable (Table 2). Lastly, the extracts of *D. cumminsii* leaf and the whole plant of *S. biafrae*, which were used as negative internal controls for the hypothesis of properties considered to be useful in choosing plant larvicides (Table 1), were inactive (Table 2). This situation was similar to that of *Euphorbia macrophylla*, used for the same purpose in an earlier report [14], and may further confirm the reliability of these four properties as factors for consideration in choosing plant larvicides.

## 3. Experimental

### 3.1. Plant Collection

The whole plant or different parts of the plants listed in Table 1 were collected from the Obafemi Awolowo University campus, Ile-Ife, Osun State or Itak Ikot Akap Ikono, Ikono Local Government Area of Akwa Ibom State after identification by the taxonomists, Prof. H.C. Illoh and Dr. (Mrs) Margaret Emmanuel Bassey, Departments of Botany, Obafemi Awolowo University, Ile-Ife and University of Uyo, Uyo, respectively. Voucher specimens were deposited in their herbaria.

### 3.2. Plant Extraction

The leaves and seeds were air-dried while the whole plants, stems and rhizomes were oven dried at 40 °C. They were subsequently powdered and 500 g of each plant material was extracted in methanol (2 L) at room temperature for 3 days, with agitation. The extract was filtered and concentrated *in vacuo*. This process was repeated two times and the combined dried extract for each plant part [45] was kept in the refrigerator (4 °C) until needed for the larvicidal assay [16].

### 3.3. Larvicidal Test

The eggs of *A. aegypti* collected from the National Medical Centre, Yaba, Lagos, Nigeria were suspended in water for 24–48 h to hatch. The larvae were fed with rabbit pellets (Bendel feeds, Edo State), until they reached the 4th instars stage. Larvicidal tests were done using slight modification [16] of the standard method [15]. Stock solutions (25 mg/mL) of the extracts were prepared by solubilising in dimethylsulphoxide (DMSO) and diluted with distilled water to give a 0.02% final concentration of DMSO. They were thereafter serially diluted to obtain 25 mL of different concentrations (0–5 mg/mL) of the test agents and twenty five larvae were introduced into each cup. The toxicity of endosulphan, a commercial insecticide, was evaluated as the positive control at 0.312, 0.625, 0.937, 1.25, 1.56 and 1.88 mg/mL. Mortality was recorded after 24 and 48 h of exposure during which no nutritional supplement was added [15]. The mean and standard error of the mean for six replicates were calculated while the percentage mortalities, LC_50_ and LC_90_ values, representing the concentrations for 50 and 90% larval mortalities, were predicted using Microsoft Excel program 2007 [16]. No mortality was observed with the negative control.

### 3.4. Statistical Analysis

The larvicidal activities of the extracts were compared with that of Endosulphan using one way analysis of variance (ANOVA) followed by Student-Newmann-Keul *post-hoc* test [14]. *p* < 0.05 was considered as significant.

## 4. Conclusions

A 61% of the eighteen extracts of the plants either used ethnomedically or reported to have antimalarial, febrifugal; insecticidal and insect repellent activities demonstrated high to moderate larvicidal activity, in agreement with Adebajo *et al.*’s [14] hypothesis of using these factors to source plant larvicides. Extracts of *P. nigrum* and *A. precatorius* seeds were the most active and would be interesting to determine the larvicidal constituent(s) of the latter.
